# Distinct effects of two hearing loss–associated mutations in the sarcomeric myosin MYH7b

**DOI:** 10.1016/j.jbc.2023.104631

**Published:** 2023-03-22

**Authors:** Lindsey A. Lee, Samantha K. Barrick, Ada E. Buvoli, Jonathan Walklate, W. Tom Stump, Michael Geeves, Michael J. Greenberg, Leslie A. Leinwand

**Affiliations:** 1 Molecular, Cellular, and Developmental Biology Department, Boulder, Colorado, USA; 2 BioFrontiers Institute, Boulder, Colorado, USA; 3 Department of Biochemistry and Molecular Biophysics, Washington University School of Medicine, St Louis, Missouri, USA; 4 School of Biosciences, University of Kent, Canterbury, United Kingdom

**Keywords:** molecular motor, myosin, actin, kinetics, SRX (super-relaxed state), myopathy, coiled-coil

## Abstract

For decades, sarcomeric myosin heavy chain proteins were assumed to be restricted to striated muscle where they function as molecular motors that contract muscle. However, MYH7b, an evolutionarily ancient member of this myosin family, has been detected in mammalian nonmuscle tissues, and mutations in MYH7b are linked to hereditary hearing loss in compound heterozygous patients. These mutations are the first associated with hearing loss rather than a muscle pathology, and because there are no homologous mutations in other myosin isoforms, their functional effects were unknown. We generated recombinant human MYH7b harboring the D515N or R1651Q hearing loss–associated mutation and studied their effects on motor activity and structural and assembly properties, respectively. The D515N mutation had no effect on steady-state actin-activated ATPase rate or load-dependent detachment kinetics but increased actin sliding velocity because of an increased displacement during the myosin working stroke. Furthermore, we found that the D515N mutation caused an increase in the proportion of myosin heads that occupy the disordered-relaxed state, meaning more myosin heads are available to interact with actin. Although we found no impact of the R1651Q mutation on myosin rod secondary structure or solubility, we observed a striking aggregation phenotype when this mutation was introduced into nonmuscle cells. Our results suggest that each mutation independently affects MYH7b function and structure. Together, these results provide the foundation for further study of a role for MYH7b outside the sarcomere.

Muscle myosin-II family members are among the most well-studied proteins in biology, and their expression and function are overwhelmingly understood to be restricted to muscle tissues. Thus, it is surprising that mutations in a sarcomeric myosin are linked to hereditary hearing loss with no apparent impacts on muscle (1). Over 1000 disease-associated mutations that cause a broad range of myopathies have been found in muscle myosin-II family members (2). However, mutations mapped to the less-studied sarcomeric myosin, MYH7b, are the first linked to hearing loss rather than a cardiac myopathy or skeletal myopathy. Exome sequencing of three siblings with severe sensorineural hearing loss revealed that all three patients were compound heterozygous for two mutations in MYH7b (1). These individuals inherited a D515N mutation in the catalytic motor domain from their mother and an R1651Q mutation in the structural rod domain from their father (Fig. 1*A*). By contrast, both parents are heterozygous for one mutation and have unaffected hearing (1). These mutations at highly conserved residues do not map to homologous positions of mutations in other known sarcomeric myosins and thus have an unknown effect on myosin function.

**Figure 1:**
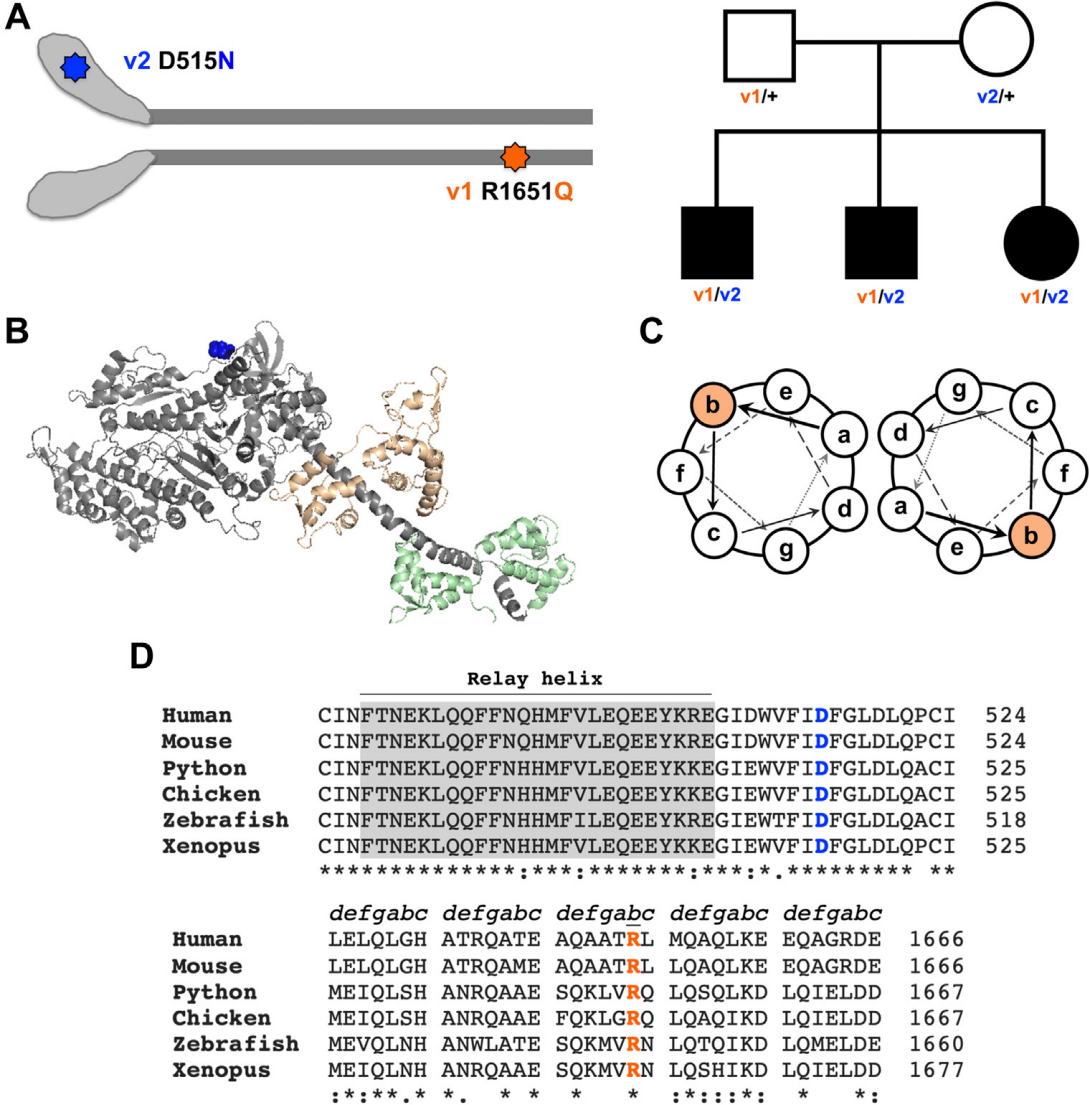
**Mutations in MYH7b are linked to individuals with hereditary hearing loss.***A, left,* schematic of the MYH7b mutation locations: D515N is found in the myosin motor domain (*blue*), and R1651Q is found in the myosin rod domain (*orange*). *Right,* a patient pedigree chart adapted from Haraksingh *et al.* (1) showing the inheritance pattern of the MYH7b mutations (*filled in boxes* and *circles* represent affected individuals). *B,* the D515 residue (shown in *blue*) mapped onto the β-MyHC structure using HBCprestroke.pdb from http://spudlab.stanford.edu/homology-models and using human MYH7b numbering to show approximate location of the motor domain mutation. The myosin heavy chain is shown in *gray*, the essential light chain (ELC) is depicted in *orange*, and the regulatory light chain (RLC) is depicted in *green*. *C,* a diagram of a coiled-coil structural motif showing amino acids within each heptad repeat at positions a – g. The R1651Q mutation is located at the outer “b” position in the heptad repeat (shown in *orange*). *D,* sequence alignments show high conservation of residues D515 and R1651 across species.

Sensorineural hearing loss can result from impairments in the inner ear structures (including the cochlea and vestibular system) and cranial nerves (3). These inner ear structures contain hair cells with actin-filled sensory stereocilia that transduce sound into electrical activity, which is propagated through the nervous system and ultimately to the brain *via* afferent neurons (4). Members of the myosin superfamily including myosin-I, myosin-III, myosin-VI, myosin-VII, and myosin-XV have roles in the organization and maintenance of the inner ear stereocilia actin-based projections, and when mutated, contribute to diseases associated with hearing loss (4, 5, 6). Phylogenetic analysis classifies MYH7b as a member of the sarcomeric myosin-II family (7), which are structurally and functionally distinct from other members of the myosin superfamily based on their long α-helical coiled-coil tail and propensity to form bipolar thick filaments. Until now, sarcomeric myosin-II family members were not implicated in inner ear function or hearing loss.

In mammals, MYH7b has a unique expression pattern because of an alternative splicing event that induces exon skipping and prevents protein production in the heart and most skeletal muscles (8). The exon skipping event is tissue specific and does not occur in certain specialized muscles that produce MYH7b protein including the extraocular muscles, muscle spindles, and upper esophagus (9, 10). *MYH7b* transcripts are abundant across a broad range of tissues, including nonmuscle (11). Exon skipping analysis revealed that transcripts able to encode MYH7b protein are present in the human brain (8), which is consistent with the recent identification of a role for MYH7b in regulating dendritic synapse structure and function in rat hippocampal neurons (12). Moreover, a literature search through high-resolution human brain mass spectrometry studies identified low levels of MYH7b protein with minor enrichment in the sensory cortex (13, 14, 15). Finally, *MYH7b* transcripts are found in the mouse inner ear cochlea, utricle, and saccule as well as the cochlear spiral ganglion neurons (16, 17). These observations and the discovery of hearing loss–associated mutations in MYH7b represent a potential unexplored role for MYH7b in nonmuscle cells and tissues.

We previously reported that MYH7b null mice lack an obvious cardiac phenotype (18), and MYH7b null mice generated by the International Mouse Phenotyping Consortium do not have any hearing defects detected by auditory brainstem response testing (19). However, loss-of-function approaches do not always model disease phenotypes, and the compound heterozygous genotype could have different effects than a null genotype. Moreover, the mutations do not appear to have a dominant-negative effect because each heterozygous parent has unaffected hearing. Rather, the combination of mutations in the motor domain and rod domain appears necessary for the hearing loss phenotype in patients. In muscle, myosin-II is a heterohexameric complex consisting of two myosin heavy chains (MyHCs), each bound by two nonidentical light chains. The MyHC consists of a catalytic motor domain that is responsible for ATPase activity and actin binding, a lever arm that amplifies small conformational changes within the motor domain to produce force against actin, and the structural rod domain, which forms a coiled-coil and drives assembly into filaments. The locations of the hearing loss mutations in the catalytic motor domain (D515N) and structural rod domain (R1651Q) suggest they are functionally distinct (Fig. 1, *B* and *C*). Both residues are highly conserved across species (Fig. 1*D*). The motor domain mutation D515N falls seven residues from the relay helix of the myosin motor domain, a region that connects the active site to the converter and is essential for force generation (20). The myosin rod forms a coiled-coil based on interactions between α-helices because of the heptad repeat (abcdefg)_n_ along the rod. The inner seam of the coiled-coil (positions a and d) is typically populated by hydrophobic residues, whereas the outer positions mediate assembly into the thick filament through charged interactions, including the “b” position where R1651 is found (Fig. 1*C*) (21). Given the surprising nature of hearing loss–associated mutations in a sarcomeric myosin, we investigated the functional impact of these mutations on MYH7b properties from the molecular to the cellular level.

## Results

### The MYH7b D515N mutation has no effect on myosin kinetics but increases actin sliding velocity and the proportion of DRX myosin

**Figure 2:**
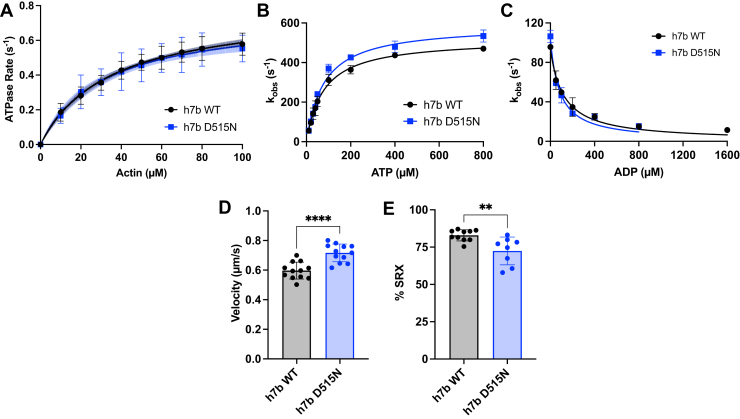
**The MYH7b motor domain D515N mutation shows distinct functional changes.***A,* actin-activated ATPase curves of h7b WT S1 compared with h7b D515N S1 show no difference in activity. Each plot shows the average of all technical replicates, and error bars represent SD. Data were fit to a Michaelis–Menten kinetics equation to obtain the *k*_cat_ and *K*_*M*_ values summarized in Table S1. The curve fit is represented by a *solid line* with shading to indicate the 95% confidence intervals. The average of ATPase curves run on the same day represent technical replicates (n = 9), and six different biological purifications are represented. *B,* ATP-induced dissociation of myosin S1 from actin plotted as observed rates against ATP concentration. The data were fit to a hyperbolic equation and yield the ATP-induced actomyosin dissociation rate (*k*_+2_) and the ATP-binding affinity (1/*K*_1_). There is no significant difference in ATP-induced dissociation rates between h7b WT and h7b D515N (n = 2). *C,* actomyosin S1 affinity for ADP plotted as observed rate constants against ADP concentration (final ATP concentration was 20 μM) and fit to a hyperbolic equation. The reaction rate constants were similar between h7b WT and h7b D515N (n = 2). Data for *B* and *C* represent mean ± SD and are summarized in Table S2. *D,* quantification of *in vitro* motility velocities of h7b WT S1 and h7b D515N S1 show an increase in actin sliding velocity because of the hearing loss mutation. The average velocity of each technical replicate (each motility video with at least eight filaments tracked represents one technical replicate, n = 12) is graphed with error bars representing SD. Data were collected for at least three independent protein purifications (biological replicates). Data are summarized in Table S3. ∗∗∗∗*p* < 0.0001. *E,* quantification of percent SRX for h7b WT S1 and h7b D515N S1 shows that the hearing loss mutation decreases the SRX proportion. Data points are technical replicates (h7b WT n = 10, h7b D515N n = 8) representing at least three biological replicates, and error bars represent SD. Data are summarized in Table S4. ∗∗*p* < 0.01. All data for h7b WT were previously reported (22). SRX, super-relaxed state.

To study the effects of the D515N mutation on motor activity, we generated recombinant human MYH7b subfragment 1 (S1; motor domain, Fig. S1*A*) constructs for WT human MYH7b (h7b WT) and the human MYH7b D515N mutant (h7b D515N). All data generated for h7b WT S1 were previously reported (22). One of the fundamental parameters of intrinsic myosin activity is the steady-state actin-activated ATPase rate, which is related to the length of time myosin takes to complete one chemomechanical cycle. We measured this rate as a function of actin concentration and determined Michaelis–Menten parameters, including maximum actin-activated ATPase rate (*k*_cat_) and apparent actin affinity (*K*_*M*_). We found no difference between h7b WT and h7b D515N maximum actin-activated ATPase rates (0.80 ± 0.08 s^−1^ and 0.78 ± 0.15 s^−1^, respectively) or apparent actin affinities (35.9 ± 9.8 μM and 38.7 ± 27.4 μM, respectively), indicating that the mutation does not affect timing of the chemomechanical cycle (Fig. 2*A* and Table S1).

Stopped-flow experiments were performed to understand whether there are any differences in the kinetics of key steps of the chemomechanical cycle. We measured the ATP-induced dissociation of myosin S1 from pyrene-labeled actin and plotted the observed rates (*k*_obs_) against ATP concentration (Fig. 2*B* and Table S2). The second-order ATP-binding rates (K_1_k_+2_) calculated for h7b WT and h7b D515N were not significantly different (6.7 ± 1.4 μM^−1^ s^−1^ and 7.6 ± 0.6 μM^−1^ s^−1^, respectively), consistent with the ATP-binding affinities, 1/*K*_1_, being similar (79.8 ± 17.7 μM and 78.5 ± 12.5 μM, respectively). Likewise, there was no significant difference in the maximum ATP-induced actomyosin dissociation rates, *k*_+2_, for h7b WT (520.7 ± 7.0 s^−1^) and h7b D515N (590.2 ± 46.6 s^−1^). Finally, we determined the ADP affinity for each construct by mixing increasing concentrations of ADP with a fixed ATP concentration and actomyosin S1, which results in a competition between ATP and ADP for the nucleotide binding site of myosin S1. Plotting the *k*_obs_ against the ADP concentration provides a measure of ADP affinity (*K*_ADP_). There was no significant difference in *K*_ADP_ between h7b WT (120.7 ± 29.5 μM) and h7b D515N (81.3 ± 19.5 μM) (Fig. 2*C* and Table S2). Next, we measured actin sliding velocity using an *in vitro* motility assay where actin movement by myosin is visualized within a flow cell. Interestingly, h7b D515N (0.716 ± 0.060 μm/s) had an increased actin sliding velocity compared to h7b WT (0.597 ± 0.057 μm/s, *p* < 0.0001) (Fig. 2*D*, Table S3, Movies S1 and S2).

Recent focus in the myosin field has centered upon the dynamic equilibrium of myosin functional states. Myosin unbound from actin can exist in two distinct functional states: the disordered-relaxed state (DRX) characterized by a slow basal ATPase rate, and the energy conserving super-relaxed state (SRX) defined by an ultra-slow ATPase rate (23). Our previous work showed that MYH7b has a high proportion of myosin heads in the SRX state compared with more conventional sarcomeric motors, and subsequent molecular dynamics simulations predicted that the MYH7b motor domain stabilizes the structural autoinhibited interacting heads motif (22). We next assessed whether the D515N mutation affects the SRX/DRX ratio of MYH7b. We found a decrease in percent SRX in h7b D515N compared with h7b WT (72.4 ± 9.3% and 82.9 ± 3.7%, respectively, *p* = 0.005) (Figs. 2*E* and S2 and Table S4). This change suggests that the mutation destabilizes the SRX state allowing more myosin to become available and enter the actin-bound state.

### h7b D515N has a larger step size but similar detachment kinetics compared with h7b WT

**Figure 3:**
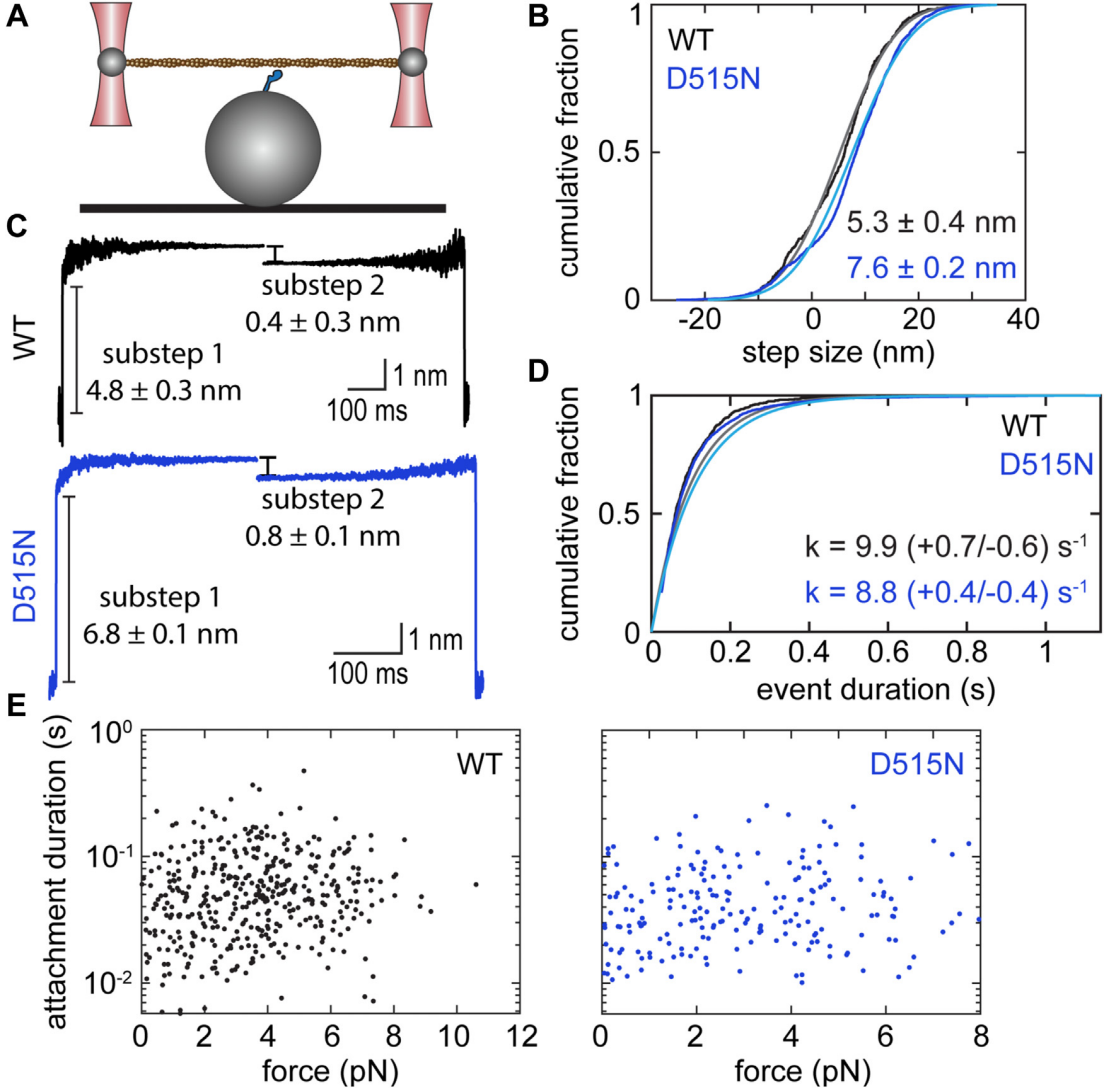
**Mechanical and kinetic characterization of h7b WT and h7b D515N by optical trapping.***A,* schematic of three-bead optical trapping assay in which an actin filament is suspended between two optically trapped beads and lowered on to a surface bead that is sparsely coated with myosin. *B,* cumulative distribution of total step sizes observed for WT (*black data trace*, *gray fit line*) and D515N (*blue data trace*, *cyan fit line*) MYH7b S1. Step sizes are reported as mean ± SEM. h7b D515N has a longer working stroke than h7b WT (*p* < 0.001). *C,* ensemble averages of individual binding events for h7b WT (*top*, *black*) and h7b D515N (*bottom*, *blue*) showing a two-substep working stroke. The first substep is significantly larger for h7b D515N relative to h7b WT (*p* < 0.001), whereas we did not observe a change in the size of the second substep (*p* = 0.26). *D,* cumulative distribution of binding interaction durations observed for h7b WT (*black data trace*, *gray fit line*) and h7b D515N (*blue data trace*, *cyan fit line*) at 1 μM ATP. The detachment rates of h7b WT and h7b D515N are similar (*p* = 0.39). *E,* attachment durations as a function of force measured for h7b WT (*left*, *black*) and h7b D515N (*right*, *blue*). Each point represents an individual binding interaction. Data were fitted with Bell’s equation to obtain *k*_0_ (the rate of the primary force–sensitive transition) and d (the distance to the transition state), neither of which were significantly different for h7b D515N relative to h7b WT (*p* = 0.73 and 0.08, respectively). Data in *B*–*D* were previously reported in Ref. (22). S1, subfragment 1.

Given the increase in actin sliding velocity, we next sought to determine whether the D515N mutation alters the mechanical or kinetic properties of the MYH7b working stroke. We used a single-molecule optical trapping assay (Fig. 3*A*) based on the three-bead geometry (24) to measure the size of the working stroke, detachment kinetics, and force sensitivity of actomyosin detachment (25, 26) for h7b WT and h7b D515N. In this assay, actomyosin interactions are detected by bringing an actin filament suspended between two optically trapped beads close to a surface bead that is sparsely decorated with myosin S1.

First, we measured the size of the working stroke and the detachment kinetics of h7b WT and h7b D515N S1 at subsaturating ATP concentrations (1 μM ATP) to facilitate the observation of binding interactions. It should be noted that under these conditions, the rate of actomyosin dissociation is limited by the rate of ATP binding rather than the rate of ADP release. As previously reported (22), we observed a total step size for h7b WT of 5.3 ± 0.4 nm (mean ± SEM) (Fig. 3*B*), which is consistent with those previously observed for human and porcine β-cardiac myosin (∼5–7 nm) (25, 26, 27, 28, 29, 30, 31). The D515N mutation resulted in an increased step size of 7.6 ± 0.2 nm (*p* < 0.001) (Fig. 3*B*).

We also examined the substeps of the MYH7b working stroke for both h7b WT and h7b D515N using ensemble averaging of individual binding events (25, 32). Ensemble averaging can reveal substeps that are obscured by Brownian motion–induced fluctuations in single binding interactions. Our data show that like many myosin isoforms, both h7b WT and h7b D515N undergo a two-substep working stroke (26, 33, 34, 35, 36, 37) (Fig. 3*C*). The data demonstrate that the increased step size observed for h7b D515N is due to an increase in the displacement generated during the first substep (6.8 ± 0.1 nm for h7b D515N *versus* 4.8 ± 0.3 nm for h7b WT, *p* < 0.001), whereas we do not detect a statistically significant change in the size of the second substep (0.8 ± 0.1 nm for h7b D515N *versus* 0.4 ± 0.3 nm for h7b WT, *p* = 0.26) (Fig. 3*C*).

The actomyosin detachment rate at 1 μM ATP can be determined from the observed distribution of attachment durations. We observed similar detachment rates for h7b WT (9.9 [+0.7/−0.6] s^−^)^1^ and h7b D515N (8.8 [+0.4/−0.4] s^−1^; *p* = 0.39) (Fig. 3*D*). Thus, the rate of ATP-induced actomyosin dissociation measured in the optical trap is similar for h7b WT and h7b D515N, consistent with our stopped-flow kinetics results. As noted previously, these rates were measured at a low ATP concentration, in contrast to the physiological condition of saturating ATP concentrations.

In order to measure the binding kinetics at saturating ATP concentrations and to examine the effects of load on myosin mechanics, we used an isometric optical clamp. In this configuration, the position of one optically trapped bead (the motor bead) is continuously adjusted by a feedback loop to keep the position of the other trapped bead (the transducer bead) constant (26, 38). Upon actomyosin binding, the motor bead exerts force on the myosin that opposes the force generated by the myosin working stroke. For each binding event, the attachment duration and the load on the myosin are measured. As can be seen from the data, the attachment duration increases gradually as force is increased for both WT and mutant myosins (Fig. 3*E*). This force-induced slowing of actomyosin dissociation has been observed for many myosin isoforms, including β-cardiac myosin (26).

To quantify this behavior, maximum likelihood estimation was used to determine the rate of the primary force–sensitive transition, *k*_o_, and the distance to the transition state, *d*, a measurement of the force sensitivity where a larger *d* denotes higher force sensitivity (see the Experimental procedures section for details). We did not detect a difference in the rate of the primary force–sensitive transition, *k*_o_, between h7b WT (25 [−3/+4] s^−1^ [mean and associated 95% confidence interval]) and h7b D515N (24 [−5/+7] s^−1^, *p* = 0.73). Importantly, the primary force–sensitive transition at saturating ATP is the same transition that limits actomyosin dissociation in the absence of force, and therefore, our data demonstrate that h7b WT and h7b D515N have similar detachment rates at saturating ATP. Moreover, we see no difference for the distance to the transition state, d, between h7b WT (0.4 [−0.1/+0.1] nm) and h7b D515N (0.1 [−0.2/+0.3] nm, *p* = 0.08). This distance to the transition state is similar to that previously observed for β-cardiac myosin (26, 39).Thus, we do not detect any mutation-induced differences in the force-dependent mechanics of MYH7b at physiologically relevant saturating ATP concentrations. Taken together, these data demonstrate that the D515N mutation increases the size of the working stroke without affecting the detachment kinetics at either saturating or subsaturating ATP concentrations.

### The R1651Q rod domain mutation has no effect on secondary structure or solubility

**Figure 4:**
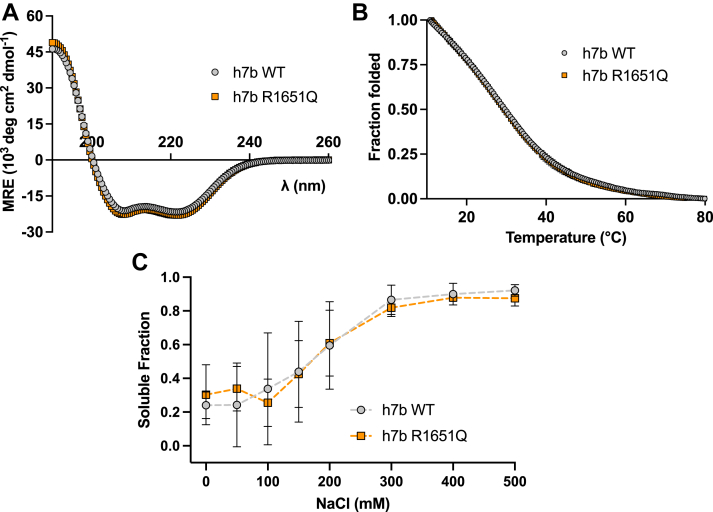
**The MYH7b rod domain R1651Q mutation has no effect on secondary structure or solubility.***A,* CD spectra for h7b WT and h7b R1651Q 15-heptad repeat constructs show no change in secondary structure as measured by mean residue ellipticity (MRE) at 10 °C. Data represent mean ± SD, n = 3. *B,* average thermal melt curves for h7b WT and h7b R1651Q show no changes in thermal stability. Data represent mean ± SD, n = 2. The fraction folded was calculated by normalizing to 100% folded at 10 °C and 0% folded at 80 °C. *C,* solubility curves for COS-7 cell lysates transfected with GFP-tagged h7b WT or h7b R1651Q rod constructs show no difference in the proportion of myosin in the soluble fraction at differing NaCl concentrations. A two-way *t* test determined no statistical difference at each concentration. Data show mean ± SD, n = 4 separate transfections.

We next determined the effects of the R1651Q mutation on the structural and assembly properties of the myosin rod. In order to understand the effects of the R1651Q mutation, we used a construct encompassing 15-heptad repeats of the rod domain with the mutation centered in the middle heptad repeat (as established by Ref. (40), Fig. S1*B*). Circular dichroism (CD) measurements on our proteins showed a spectral signal characteristic of an α-helix with minima at ∼208 and 222 nm (Fig. 4*A*) (41). The h7b R1651Q CD spectrum overlapped with that of h7b WT, indicating no effect on α-helical structure (Fig. 4*A*). Similarly, the R1651Q mutation did not change a melting curve measured at 222 nm from 10 to 80 °C, indicating that the mutation does not affect thermal stability (Fig. 4*B*).

Given that the R1651Q mutation is found in the “b” position of the heptad repeat on the outer surface of the coiled-coil, we hypothesized that this mutation may affect coiled-coil assembly properties that drive filament formation. Myosin forms insoluble filaments at low ionic strengths, and these filaments disassemble in high salt because of disruption of salt bridges (42). We transfected nonmuscle COS-7 cells with a GFP-tagged myosin rod construct encoding amino acids 847 to 1941 of h7b WT or h7b R1651Q, and after 24 h, lysed the cells in increasing salt concentrations. We then quantified the soluble fraction at each salt concentration using a fluorescence readout to identify any changes in solubility between h7b WT and h7b R1651Q. We observed a characteristic trace where the myosin rod becomes more soluble at high salt, but there was no difference in solubility between the h7b WT and h7b R1651Q mutant (Fig. 4*C*). These results suggest that the R1651Q mutation does not affect the secondary structure or the salt-dependent solubility profile of the myosin rod.

### The R1651Q rod domain mutation causes aggregate formation in cells

**Figure 5:**
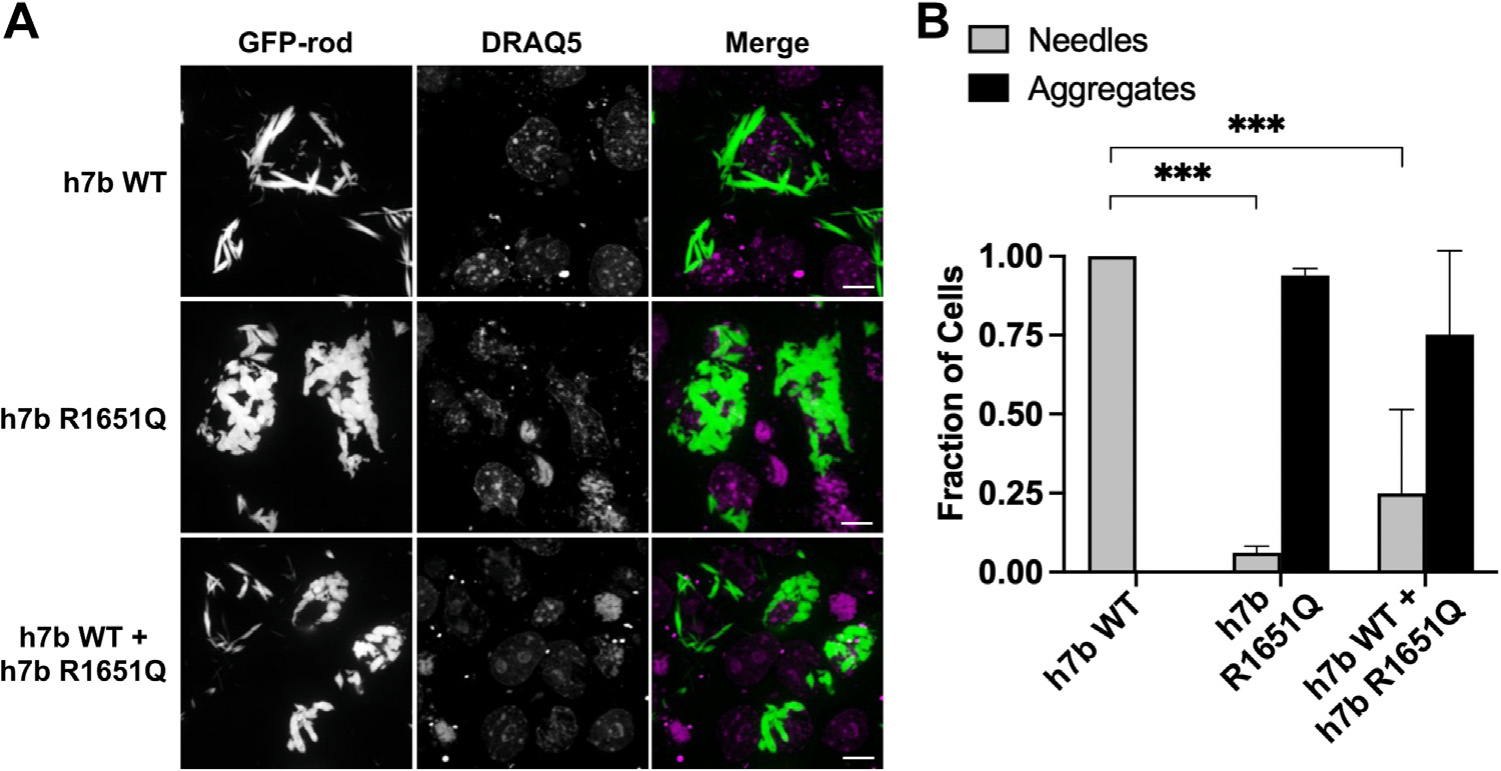
**The MYH7b R1651Q mutation alters filament formation in COS-7 cells.***A,* COS-7 cells transfected with GFP-myosin rod constructs encoding amino acids 847 to 1941. Representative images for h7b WT alone (*top panel*), h7b R1651Q alone (*middle panel*), and h7b WT + h7b R1651Q (*bottom panel*) are shown. All images were taken 18 to 22 h after transfection. Cells were stained with DRAQ5 nuclear stain to identify individual cells. Scale bar represents 10 μm. *B,* scoring COS-7 cells according to the phenotypes: “needles” (as seen in h7b WT cells) or “aggregates” (as seen in the h7b R1651Q transfection). The number of cells with each phenotype was scored blinded (Table S5), and the bar graph shows quantification (mean ± SD) for at least three separate images for each transfection (h7b WT alone, h7b R1651Q alone, or h7b WT + h7b R1651Q). Analysis with one-way ANOVA shows a significant difference in cell phenotype between h7b WT and h7b R1651Q and h7b WT + h7b R1651Q but no difference between h7b R1651Q alone and h7b WT + h7b R1651Q). ∗∗∗*p* < 0.001.

We next assessed self-assembly properties of the h7b WT and h7b R1651Q MYH7b rod using a previously established assay where sarcomeric myosin filament formation can be tracked in nonmuscle cells (43, 44, 45, 46). We transfected COS-7 cells with an N-terminally GFP-tagged myosin rod construct and after 18 to 22 h, used live-cell confocal microscopy to visualize filament formation. Cells transfected with the h7b WT construct formed long and pointed needle-like filaments within the cell cytoplasm (Fig. 5*A*, *top row*). Strikingly, cells transfected with the h7b R1651Q rod mutation formed rounded blob-like aggregates with shorter and more rotund filaments (Fig. 5*A*, *middle row*). Cell scoring revealed that almost 100% of cells transfected with this construct developed this aggregate phenotype (Fig. 5*B* and Table S5). In the family affected by hearing loss, the parent with the rod domain mutation is heterozygous, therefore we cotransfected h7b WT and h7b R1651Q in a 1:1 ratio to mimic this condition and determine if the WT allele rescues the aggregate phenotype (Fig. 5*A*, *bottom row*). Although we observed a slight increase in the number of cells scored as the h7b WT needle-like phenotype, there was not a significant phenotypic rescue over the h7b R1651Q-alone transfection (Fig. 5*B* and Table S5). Because both constructs are GFP tagged, we cannot differentiate whether the filaments contain heterodimers of h7b WT and h7b R1651Q. However, past studies carried out with dual-labels demonstrated the formation of heterodimers when cotransfecting cells with two different rod constructs (43, 44).

We observed similar results transfecting nonmuscle COS-7 cells with GFP-tagged full-length constructs containing h7b WT, h7b R1651Q, or a 1:1 mixture of h7b WT and h7b R1651Q (Fig. S3, columns 1–3). We also transfected full-length MYH7b constructs containing the D515N motor domain mutation or a double D515N/R1651Q mutation (Fig. S3, columns 4 and 5). The D515N-containing construct produced filaments similar to the h7b WT construct, and the double D515N/R1651Q construct produced aggregates similar to the h7b R1651Q mutation–alone construct. The conclusions drawn from these full-length transfections with respect to the motor domain are limited because COS-7 cells do not contain the muscle-specific chaperones necessary to properly fold the myosin motor domain. However, in a muscle context using neonatal rat ventricular myocytes (NRVMs) electroporated with the same WT or mutant constructs, the D515N motor domain mutation has no discernable effect on sarcomere structure (Fig. S4). The R1651Q mutation causes aggregate formation in NRVMs similar to those previously observed, which disrupt sarcomere organization (Fig. S4). Overall, this pervasive aggregation phenotype suggests that the R1651Q mutation causes defects in myosin rod assembly.

## Discussion

In this study, we sought to further elucidate the biological role of MYH7b by examining the molecular and cellular impacts of the two MYH7b hearing loss–associated mutations, D515N and R1651Q. While expression of canonical sarcomeric myosin-II family members is restricted to striated muscle tissue, and pathogenic mutations therefore primarily impact heart or skeletal muscle function, MYH7b is atypical in both respects. Thus, there is correspondingly little known about its biological role. In an effort to expand our knowledge of MYH7b function, we took advantage of several observations. First, MYH7b protein is expressed at low levels in certain human nonmuscle tissues, including the brain (13, 14, 15). In fact, silencing of *MYH7b* RNA in neurons results in morphological and functional defects in neurons (12). Second, two MYH7b mutations are associated with hearing loss with no overt muscle phenotype (1). Third, despite differences in apparent biological function, the MYH7b sequence is clearly homologous to sarcomeric myosins (7). In addition, our data show that the MYH7b motor domain is an actin-activated ATPase that has properties consistent with a role in force generation. Therefore, even though there are no sarcomeres in tissues comprising the auditory system, it was reasonable for us to test whether hearing loss–associated mutations affect enzymatic and structural properties of MYH7b.

We found that the D515N motor domain mutation resulted in certain gain-of-function impacts. Although actin-activated ATPase activity and detachment kinetics were unchanged, the mutation moderately but significantly increased actin sliding velocity. The sliding velocity is proportional to the step size times the detachment rate (*i.e.*, 1/attachment duration) at saturating ATP (47). Given that we did not measure any increases in detachment rate in the kinetic or optical trapping assays, the increase in actin sliding velocity is most likely accounted for by the larger step size measured by optical trapping. It is worth noting that several disease-associated mutations in the closely related β-MyHC, including the well-characterized hypertrophic cardiomyopathy–associated R403Q mutation cause increases in actin sliding velocity (28, 29). We also found that the MYH7b motor has a similar force sensitivity to β-cardiac myosin, suggesting that this motor likely functions in power generation rather than anchoring. Finally, we observed that the D515N mutation increases the proportion of DRX myosin compared with h7b WT, another disease mechanism commonly observed for hypertrophic cardiomyopathy–causing mutations (48, 49, 50). Future work using two-headed myosin constructs sufficient to form the autoinhibited interacting heads motif state will be necessary to understand whether structural perturbations arising from the D515N mutation contribute to the observed decrease in SRX population. Considering these results together, it is plausible that the changes in motor activity because of the D515N mutation contribute to the hearing loss phenotype seen in patients. Of note, the recombinant myosin S1 proteins used in this study are bound by endogenous mouse C_2_C_12_ myosin light chains. Myosin light chain isoforms can influence a myosin’s motor properties (51, 52, 53), so future studies to identify native myosin light chain–binding partners of MYH7b will be critical for improving our understanding of MYH7b function in nonmuscle and in health and disease states. Even so, in this study, both h7b WT and h7b D515N recombinant proteins are bound by the same mouse light chains and thus the functional changes measured are specifically driven by the D515N mutation.

The R1651Q rod domain mutation changes a positively charged residue to a polar uncharged group in the exposed “b” position of the heptad, which likely mediates charge interactions between coiled-coils (21). Therefore, we analyzed several myosin structural properties. Unsurprisingly, given the topographical location of position 1651, the mutation did not impact secondary α-helical structure as assessed by CD, and there was no effect on thermal stability of the myosin rod. We also observed no impact on filament solubility in lysates from cells transfected with rod constructs, indicating that the hearing loss–associated MYH7b R1651Q mutation does not change sensitivity to ionic strength. These findings contrast with other studies of myopathy-associated myosin rod mutations that affect secondary structure or thermal stability, although the effects of these mutations appear to vary in severity (54, 55, 56, 57).

Despite finding no impact of the rod mutation on secondary structure or solubility profile, we observed a distinct and ubiquitous aggregation phenotype when the R1651Q mutation was introduced into COS-7 cells and NRVMs. Previous studies of myosin rod mutations identified aggregate or puncta formation in cells, although a much lower proportion of cells showed this phenotype compared with our results (43). The pervasiveness of the aggregate phenotype we observed suggests a potential greater pathogenicity of the R1651Q mutation. To date, no detailed molecular analysis has been performed on myosin rod missense mutations with glutamine substitutions. Notably, glutamine repeats contribute to protein aggregates that characterize many neurodegenerative diseases (58), presenting a reasonable hypothesis for the molecular pathogenicity of the MYH7b R1651Q mutation in hearing loss. Within the 15-heptad repeat region including the R1651Q mutation, glutamine is the fourth most frequent amino acid found in the sequence. Although the R1651Q substitution does not dramatically change the frequency of glutamine residues, it would be worth performing mechanistic studies in the future to understand if this single substitution causes glutamine-mediated aggregation.

The hearing loss patients showed compound heterozygosity, meaning that each copy of their *MYH7b* gene carries either the rod or motor mutation, and they do not express any WT protein. However, neither heterozygous parent showed evidence of clinical hearing loss, suggesting that the mutations may exert their effects only in the absence of any WT protein. Myosin can form homodimers or heterodimers when two or more myosin isoforms are coexpressed (43, 59). Thus, the presence of dimers containing the WT protein is a potentially important difference between the unaffected parents and the hearing-impaired children. We suggest that some combination of both the D515N and R1651Q mutations forming homodimers with themselves and heterodimers with each other are likely responsible for the hearing loss condition. Alternatively, heterozygous penetrance may not be 100%, which is the case for some disease-causing myosin mutations (60, 61). Introducing the h7b WT rod with the h7b R1651Q rod at a 1:1 ratio did not significantly rescue the aggregate phenotype, but there was a slight improvement as seen by an increase in needle-like filaments in these cells. In the parent condition, h7b WT homodimers and heterodimers of h7b WT and h7b R1651Q could be enough to overcome the deleterious effects of the R1651Q mutation *in vivo*. Furthermore, recent evidence suggests that myosin rod domain mutations can exert effects on the SRX/DRX ratio (62), and thus, the minor functional changes seen in the D515N mutation, including a slight increase in DRX, could be amplified by instability across the whole molecule imparted by the R1651Q mutation. Further studies to better understand how the two mutations may interact will be necessary to mechanistically define how these mutations together contribute to hearing loss. The work presented in this study lays the foundation for this understanding, and the observation that these hearing loss–associated mutations have distinct molecular and cellular phenotypes argues that MYH7b has a role in auditory tissues.

## Experimental procedures

The mutations described by Haraksingh *et al.* (1) were originally annotated at positions D557 and R1693 based on the 2014 human genome annotation for MYH7b, which was published with a 42-amino terminal extension that has since been corrected in the National Center for Biotechnology Information. With the current National Center for Biotechnology Information sequence (lacking the 42-amino terminal extension), the mutations map to D515 and R1651.

### DNA constructs

The full-length human MYH7b construct was cloned from the MYH7b complementary DNA clone pF1KA1512 (product ID FXC06072; Kazusa DNA Research Institute) into an enhanced GFP (eGFP) containing plasmid with an N-terminal EGFP tag linked to human MYH7b full-length complementary DNA sequence by a 13-amino acid polylinker (Ser-Gly-Arg-Thr-Gln-Ile-Ser-Ser-Ser-Ser-Phe-Glu-Phe). The MYH7b rod construct, amino acids 847 to 1941, was amplified from the full-length MYH7b sequence and subcloned into the pEGFP plasmid where N-terminal eGFP is directly linked to the MYH7b rod sequence (no polylinker). The D515N and R1651Q mutations were introduced to the MYH7b full-length clones using synthetic constructs encoding the mutations (Genewiz). The MYH7b 15 heptad repeat constructs centered on heptad 29 containing residue R1651 (amino acids 1597–1701) were made by amplifying sequences from the MYH7b WT and MYH7b R1651Q rod constructs and subcloning into pGEX-4T-1 vector (MilliporeSigma; catalog no.: GE28-9545-49). The resulting plasmid has an N-terminal glutathione-*S*-transferase (GST) tag linked to MYH7b WT or R161Q 15 heptad repeat through a thrombin-Gly-Ser-Gly-Ser-Gly linker. Finally, recombinant human MYH7b WT and MYH7b D515N myosin constructs were generated by subcloning the S1 region (amino acids 1–850) from templates mentioned previously into a pUC19 vector containing the PDZ binding C-tag (Arg-Gly-Ser-Ile-Asp-Thr-Trp-Val). Original pShuttle-CMV constructs were later updated to include the translation enhancing WPRE sequence cloned using a synthetic construct (Genewiz) directly after the C-tag and stop codon using the Gibson HiFi DNA Assembly Cloning Kit (NEB; catalog no.: E5520S). All constructs were screened *via* sequencing for the correct sequence.

### Recombinant myosin expression and purification

Recombinant myosin was produced as described previously with minor changes (22, 27, 63, 64). Briefly, adenoviruses encoding myosin S1 were used to infect C_2_C_12_ cells 3 days after differentiation. Cells were collected 4 days after infection, pelleted, collected in liquid nitrogen, and stored at −80 °C. Cells were thawed and lysed in 50 mM Tris (pH 8.0), 200 mM NaCl, 4 mM MgCl_2_, 0.5% Tween-20, 5 mM DTT, 1 mM ATP, 0.2 mM PMSF, and 1× protease inhibitor cocktail (MilliporeSigma/Roche catalog no.: 11873580001), dounce homogenized, and then spun down at 39,000*g*. The supernatant was filtered through 5 and 1.2 μM filters and applied to a SulfoLink resin (ThermoFisher; catalog no.: 20402) coupled to PDZ. The column was washed with a buffer containing 30 mM Tris (pH 7.5), 50 mM KCl, 5 mM MgCl_2_, 1 mM DTT, and 1 mM ATP. The WETWV (Genescript) peptide was used to elute myosin S1, which was dialyzed against a storage buffer containing 20 mM Mops (pH 7.0), 25 mM KCl, 5 mM MgCl_2_, and 10% sucrose. 1 mM DTT and 1 mM ATP were added to the proteins, and they were frozen in liquid nitrogen and stored at −80 °C.

### Actin-activated ATPase assay

Actin was purified as previously described (65). Actin-activated ATPase rates were measured using an NADH-coupled ATPase assay as described previously (22). Briefly, myosin was diluted to 0.8 μM (the final assay concentration was 0.4 μM) in 20 mM Mops (pH 7.0), 25 mM KCl, and 5 mM MgCl_2_ with 5 mM DTT. About 4 μM gelsolin and actin at concentrations ranging from 10 to 100 μM were added to each well of a 384-well plate with myosin. A 10× coupling buffer made of 20 mM ATP, 30 mM phospho(enol)pyruvate (Sigma; catalog no.: 860077), 10 mM NADH (Sigma; catalog no.: N8129), and 8 mM pyruvate kinase/lactate dehydrogenase (Sigma; catalog no.: P0294) was added to the each well at a final 1× concentration. Absorbance at 340 nm was measured every 30 s for 1 h at 25 °C using a SpectraMax iD3 Multi-Mode Microplate Reader (Molecular Devices). Individual rates in each well were determined based on the linear range of absorbance *versus* time. A control well with no actin was used to calculate the basal ATPase rate and subtracted from the other well’s rates. The data were fit using a Michaelis–Menten kinetics equation in GraphPad Prism (GraphPad Software, Inc) to determine the maximum actin-activated ATPase rate (*k*_cat_) and Michaelis–Menten constant (*K*_*M*_). Ambiguous fits were discarded across all datasets, and statistical significance was determined using a two-tailed *t* test.

### *In vitro* motility assay

*In vitro* motility was performed as previously described (22). Briefly, prior to flow cell loading, a “deadheading” spin down was performed where actin and myosin (at a 3:1 M ratio) was incubated on ice for 5 min, 1 mM ATP was added to the mixture, and it was centrifuged at 90,000 rpm in a TLA-100 rotor (Beckman) for 25 min at 4 °C, and the supernatant containing active myosin was collected. 3 μM SNAP-PDZ in 1× assay buffer (20 mM Mops [pH 7.0], 25 mM KCl, 5 MgCl_2_) was flowed into a flow cell constructed of a microscope slide, double-sided tape, and coverslips coated in 0.2% nitrocellulose in amyl acetate, followed by 1 mg/ml bovine serum albumin (BSA) in 1× assay buffer, and then myosin S1 diluted to 0.4 μM in 1× assay buffer. After another blocking step with 1 mg/ml BSA, labeled rhodamine–phalloidin actin was flowed in followed by a motility buffer consisting of 1× assay buffer, 3 mM ATP, 1 mg/ml BSA, 1 mM EGTA, and 0.5% methylcellulose and an oxygen scavenging solution of 4 mg/ml glucose, 0.135 mg/ml glucose oxidase (Sigma; catalog no.: G2133), 0.0215 mg/ml catalase (Sigma; catalog no.: C30). Videos were collected at a frame rate one frame per second for 30 s at 25 °C using a 100× oil objective on a Nikon Ti-E Eclipse Inverted Fluorescence Microscope with a Hamamatsu ORCA-Flash 4.0 V3 Digital CMOS Camera. *In vitro* motility videos were analyzed using actin tracking analysis software developed in house (see Ref. (22) for details). The mean velocity across technical replicates was determined, and statistical significance was determined using a two-tailed *t* test.

### Stopped-flow kinetics

Stopped-flow assays were performed as previously described (22). Briefly, a high-tech scientific SF-61 DX stopped-flow was used with buffer conditions of 20 mM Mops (pH 7.0), 25 mM KCl, 5 mM MgCl_2_, and 1 mM DTT at 20 °C. Rabbit skeletal actin was labeled with pyrene and excited with an Hg–Xe lamp, exciting at 365 nm with the emission measure through a KV-399 cutoff filter.The data were graphed in GraphPad Prism. Statistical significance was determined using an unpaired *t* test.

### Optical trapping

#### Optical trapping experiments

Optical trapping was performed as previously described (22) using porcine cardiac actin and a custom-built microscope-free dual-beam optical trap (25). All solutions used KMg25 buffer (60 mM Mops [pH 7.0], 25 mM KCl, 2 mM EGTA, 4 mM MgCl_2_, and 1 mM DTT) unless otherwise noted. A myosin dead head ultracentrifugation step was performed before each experiment. Flow cells were sparsely coated with silica beads suspended in nitrocellulose in amyl acetate (25, 66) and then loaded with 20 nM SNAP-PDZ for 5 min, blocked with 1 mg/ml BSA for 5 min, and loaded with myosin S1 (20–60 nM) for 5 min. The surface was blocked with 1 mg/ml BSA and then loaded with activation buffer (KMg25 with 1 mg/ml BSA, 1 μM ATP, 192 U/ml glucose oxidase, 48 μg/ml catalase, 1 mg/ml glucose, and ∼25 pM rhodamine–phalloidin-stabilized actin). Finally, 4 μl of streptavidin beads in 1 mg/ml BSA was loaded, and then the flow cell was sealed with vacuum grease and data were collected within 60 min. Rhodamine–phalloidin-stabilized actin filaments (containing 15% biotinylated actin) were attached to polystyrene beads using a biotin–streptavidin linkage (25, 66). For each bead-actin-bead assembly, the trap stiffness was calculated from fitting of the power spectrum as previously described (66). Data were collected at 20 kHz and filtered to 10 kHz according to the Nyquist criterion.

#### Implementation of the isometric optical clamp feedback

Here, we used an all-digital implementation of an isometric feedback clamp (38). In an isometric optical clamp feedback experiment, the position of one bead (the “transducer”) is continuously sampled, and deviations from its original “setpoint” position are compensated for by moving the second (“motor”) bead using acoustic optical deflectors (AODs; Gooch and Housego). The positions of the beads were recorded using quadrant photodiodes, the feedback calculations were digitally performed on a field programmable gate array board (National Instruments; PCIe-7852), and the laser controlling the motor bead was translated using AODs.

The error signal used for the feedback, V_*t*_, is the time filtered positional error for the current sample period given by:

$$V_{t}=K_{p}E_{t}+K_{i}\sum_{k=t}^{t-W}E_{k}$$

where *K*_*p*_ is the user-defined proportional gain, *K*_*i*_ is the user-defined integral gain, *E*_*t*_ is the current sample’s absolute error from the setpoint, and *W* is the user-defined integration window (up to a 255-sample memory). The compensating position for the motor bead in frequency units is then calculated by the field programmable gate array and transmitted to the parallel port interface of the Digital Frequency Synthesizer (Analog Devices AD9912A/PCBZ), which controls the beam deflection angle from the AOD. The feedback loop can run at a maximal speed of 50 kHz.

The time constant for the feedback response time was set as described (38). Briefly, a bead-actin-bead dumbbell was held in the dual beam traps, a square wave was injected into the transducer bead channel, and then the movement of the motor bead by the feedback system was monitored. The proportional and integral gains were empirically adjusted to give a response time of ∼5 ms without introducing oscillations into the system.

#### Analysis of single-molecule data

All data from optical trapping experiments were analyzed using a custom-built MATLAB (MathWorks) program, SPASM (25). Step size data are reported as mean ± standard error. Statistical testing of the step sizes was done using a two-tailed Student’s *t* test of individual binding interactions. Event durations were fit by single exponential functions using the MATLAB-based program MEMLET (67) to determine the best-fit value for the detachment rate and the associated 95% confidence interval, which was determined by bootstrapping. Statistical testing of detachment rates was performed using a Mann–Whitney test of individual binding interactions.

For isometric optical clamp experiments, experiments were conducted at saturating ATP concentrations. Binding interactions were identified using a variance threshold, set by the position of the transducer bead, and the force exerted by the motor bead and the attachment duration were measured. The relationship between the force, F, and the load-dependent detachment rate, *k*(*F*) was modeled using the Bell equation (68):

$$k\left(F\right)=k_{0}*\exp\left(\frac{-F*d}{k_{B}*T}\right)$$

where *k*_0_ is the rate of the primary force–sensitive transition in the absence of force, d is the distance to the transition state, and k_B_∗T is the thermal energy. The distribution of attachment durations is exponentially distributed at each force, and therefore follows the following probability density distribution (67):

k(F,t)=k(F)∗exp(−k(F)∗t)

Maximum likelihood estimation was used to determine the most likely values of *k*_0_ and d, as previously described (26, 35, 67). A dead time of 10 ms was used to correct for missing binding interactions as previously described (67). About 95% confidence intervals for parameter values fitting were determined using 1000 rounds of bootstrapping simulations, and hypothesis testing was performed using the difference in the means and the variances of these distributions as previously described (69).

### Single ATP turnover assay

Single ATP turnover experiments were performed as previously described (22). Briefly, experiments used a CLARIOstar Plate reader with two software-controlled injectors (BMG Labtech) at 25 °C. Myosin S1 was diluted to 0.8 μM in 1× assay buffer (30 mM KAc, 10 mM Tris [pH 7.5], 4 mM MgCl_2_, 1 mM EDTA, and added to a single well of a black 384-well plate. 2′-(or-3′)-O-(*N*-Methylanthraniloyl) adenosine 5′-triphosphate, trisodium salt (mant-ATP; ThermoFisher, catalog no.: M12417) was injected into the well at twofold to eightfold excess (0.8–3.2 μM), and the plate was briefly mixed for 3 s at 300 rpm. After 60 s, excess unlabeled ATP (4 mM) was injected, and fluorescence was monitored over time (365 nm excitation/450 nm emission). Fluorescence signal *versus* time was plotted, normalized, and fit using GraphPad Prism to a biexponential decay function, which provides the rates and amplitudes of the fast (DRX) and slow (SRX) phases. Ambiguous fits were excluded across all datasets. Statistical significance was determined using a two-tailed *t* test.

### Myosin rod domain cloning, expression, and purification

#### Bacterial growth and induction

pGEX-4T-1 plasmids with MYH7b WT or MYH7b R1651Q were transformed into BL21 DE3-competent cells (NEB; catalog no.: C2527I). About 100 ml LB with ampicillin was inoculated with a single colony and incubated at 20 °C overnight with shaking. The overnight colony was diluted at 1:50 in LB plus ampicillin and incubated at 37 °C with shaking. Once the absorbance at 600 nm reached ∼0.8, the culture was induced with 0.5 mM IPTG and cold shocked on ice for 20 min. Cultures were grown for 3 h at 37 °C. Cells were harvested *via* centrifugation and washed in PBS, and the pellets were stored at −80 °C.

#### GST-purification and thrombin cleavage

Cells were resuspended in lysis buffer (50 mM Tris [pH 7.5], 150 mM NaCl, 0.1% Triton X-100, 2 M urea, 1 mg/ml lysozyme, 4 mM MgCl_2_, 5 mM DTT, 1× protease inhibitor cocktail [MilliporeSigma/Roche, catalog no.: 11873580001]) and freeze/thawed three times. Lysates were spun at 10,000*g* for 15 min, and the supernatant was filtered through 5 and 1.2 μM syringe filters onto an equilibrated column of Glutathione Sepharose 4B (MilliporeSigma; catalog no.: GE17-0756-01). After the filtered lysate flowed through, the column was washed with 50 mM Tris (pH 7.5), 150 mM NaCl, 2 mM MgCl_2_, and 1 mM DTT. GST-15H protein was eluted with 20 mM reduced glutathione in 50 mM Tris (pH 7.5) and 150 mM NaCl. Fractions were run on a gel, pooled, and dialyzed against storage buffer (20 mM Hepes [pH 7.5], 150 mM NaCl, 2 mM MgCl_2_, and 10% sucrose) or thrombin cleavage buffer (50 mM Tris [pH 8.0], 10 mM CaCl_2_, and 150 mM NaCl) overnight at 4 °C. Protein dialyzed into storage buffer was flash frozen into liquid nitrogen and stored at −80 °C. GST tag was cleaved off of proteins using the Thrombin CleanCleave Kit (MilliporeSigma; RECOMT-1KT) following manufacturer’s instructions. The cleaved protein was passed over an equilibrated column containing Glutathione Sepharose 4B, and the flow through containing the cleaved GST-free protein was collected and dialyzed into storage buffer or CD buffer overnight at 4 °C.

### CD

All CD measurements were made with 15-heptad constructs. Samples thawed on ice or freshly cleaved were dialyzed into CD buffer (50 mM NaCl, 10 mM sodium phosphate buffer [pH 7.4], and 1 mM DTT. CD spectra were measured at 10 °C from 260 to 190 nm using a modular Applied Photophysics Chirascan Plus CD Spectrometer and a 0.5 mm cuvette. Concentrations for the 15H constructs were between 150 and 200 μg/ml. Scans were performed on at least two separate protein purifications. Thermal melting measurements were taken at 222 nm and 0.5 °C increments from 10 °C to 80 °C with a 1 °C/min heating rate in CD buffer. The fraction folded was calculated by normalizing to 100% folded at 10 °C and 0% folded at 80 °C.

### Cell culture and transfection

COS-7 cells (American Type Culture Collection; catalog no.: CRL-1651) were maintained in Dulbecco’s modified Eagle’s medium with 10% fetal bovine serum, 1% l-glutamine, and 1% penicillin–streptomycin. Cells were plated on 35 mm glass bottom dishes (MatTek; catalog no.: P35G-1.5-14-C) and once 70 to 80% confluent transfected with 2 μg of DNA using TransIT-LT1 transfection reagent (Mirus; catalog no.: MIR2300). Live cells were imaged approximately 20 h later using a Nikon Spinning Disc Confocal Microscope equipped with an Andor iXon Ultra 888 detector. At least three independent transfections were performed for each construct. Live cells were stained with DRAQ5 (ThermoFisher/Invitrogen; catalog no.: 65-0880-92) at a final concentration of 1 μM before imaging. Large images were taken and blinded for cell scoring. Cells within the field of view were assigned a phenotype “aggregate” characterized by concentric, rounded, and/or curved appearance and no pointy or blunted tips, or “needles” characterized by straight lines and pointy tips. Cells that could not be differentiated from one another or had ambiguous classification were discarded from the analysis.

NRVMs were prepared as previously described (43). Isolated NRVMs (2.8 × 10^6^) were transfected with 2 μg of DNA using the Rat Cardiomyocyte-Neonatal Nucleofector Kit (Lonza; catalog no.: VPE-1002) according to the manufacturer’s instructions. Cells were plated on 35 mm glass bottom dishes (MatTek; catalog no.: P35G-1.5-14-C) for imaging as described previously.

### Solubility assay

COS-7 cells were seeded in a 6-well plate and once 60 to 80% confluency, transfected with 2 μg EGFP-MYH7b rod construct (WT or R1651Q). After 24 h, cells were scraped in 1× PBS, and equal volumes were pelleted and resuspended in 100 μl lysis buffer (0.5% Triton X-100, 50 mM Mops [pH 7.0], 5 mM EGTA, 2 mM DTT, 5 mM MgCl_2_, 5 mM ATP, and 1× protease inhibitor cocktail (Millipore Sigma/Roche; catalog no.: 11873580001) and 0.2 mM PMSF) with 0, 50, 100, 150, 200, 300, 400, or 500 mM NaCl. Cells were treated with 1 mg/ml DNaseI (EMD Millipore; catalog no.: 260913) and lysed rocking at 4 °C for 2 h. Cells were centrifuged at 140*g* for 5 min at 4 °C, and the supernatant and pellet (resuspended in equal volume at corresponding NaCl concentration) were collected. Equal volumes of pellet and supernatant as well as buffer alone were loaded onto a black and clear bottom 96-well plate, and fluorescence was measured using a SpectraMax iD3 Multi-Mode Microplate Reader at 488 nm excitation and 535 nm emission. The amount of GFP-myosin in the soluble and insoluble (pellet) fractions was calculated.

### Statistical analysis

All data except for optical trapping data were graphed and analyzed in GraphPad Prism. Unless otherwise noted, mean ± standard deviation is represented, and *p* < 0.05 was the threshold for significance. Sample size and level of statistical significance are noted in each figure legend.

## Data availability

All data are included in the article or supporting information. Raw data from this article are available upon request from the corresponding author.

## Supporting information

This article contains supporting information.

## Conflict of interest

The authors declare that they have no conflicts of interest with the contents of this article.

## Supporting information

human_MYH7b_D515N_S1_7x

human_MYH7b_WT_S1_7x
