# Supplementary material for: Distinct effects of two hearing loss–associated mutations in the sarcomeric myosin MYH7b
Source: J Biol Chem. 2023 Mar 22;299(5):104631. doi: 10.1016/j.jbc.2023.104631 (PMC10141508; doi:10.1016/j.jbc.2023.104631)
Supplement: Supporting information [file mmc3.pdf]

Supporting Information for

Distinct effects of two hearing loss-associated mutations in the sarcomeric myosin MYH7b

Lindsey A. Lee[1,2], Samantha K. Barrick,[3] Ada E. Buvoli[1,2], Jonathan Walklate[4], W. Tom Stump[3], Michael Geeves[4], Michael J. Greenberg[3], Leslie A. Leinwand[1,2]

[1]Molecular, Cellular, and Developmental Biology Department, Boulder, CO, USA.
[2]BioFrontiers Institute, Boulder, CO, USA.
[3]Department of Biochemistry and Molecular Biophysics, Washington University School of Medicine, St. Louis, MO, USA.
[4]School of Biosciences, University of Kent, Canterbury, UK

Supporting information

This PDF includes:

Figures S1-S4.
Table S1-S5.

Other supporting information for this manuscript include:

Movie S1. Actin sliding motility of human MYH7b WT S1. Playback speed 7x.

Movie S2. Actin sliding motility of human MYH7b D515N S1. Playback speed 7x.

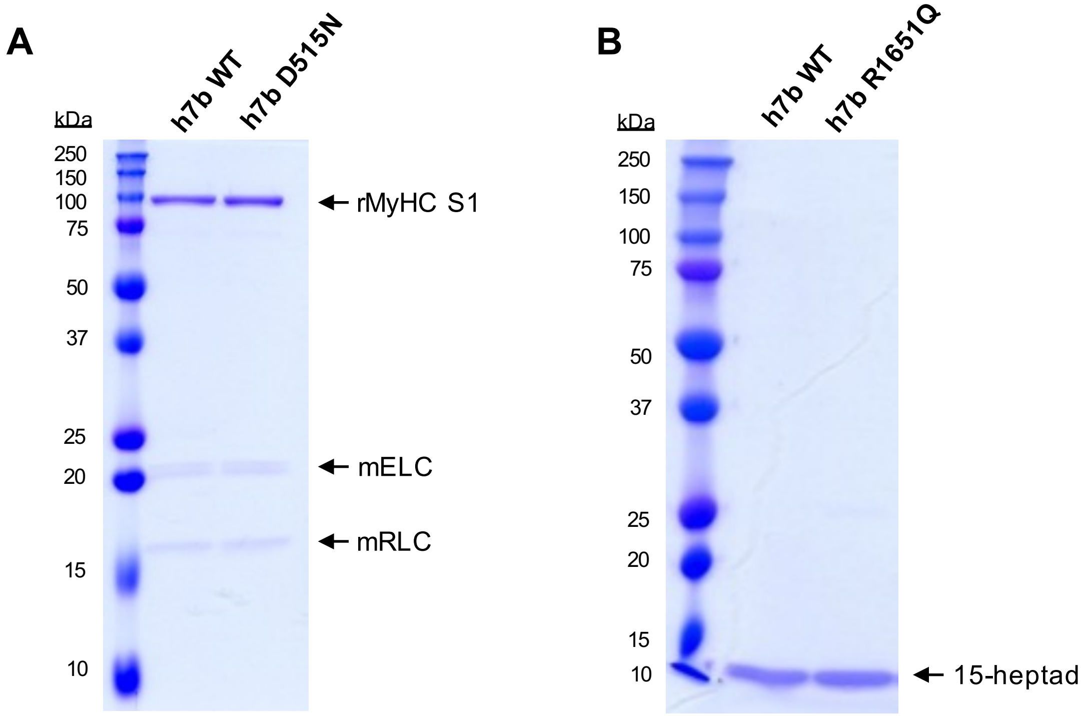

Figure S1. A. Representative gel of recombinant myosin heavy chain subfragment 1 (rMyHC S1) bound by C_2C_{12} endogenous mouse essential light chain (mELC) and mouse regulatory light chain (mRLC). **B.** Representative gel of myosin rod 15-heptad repeat constructs.

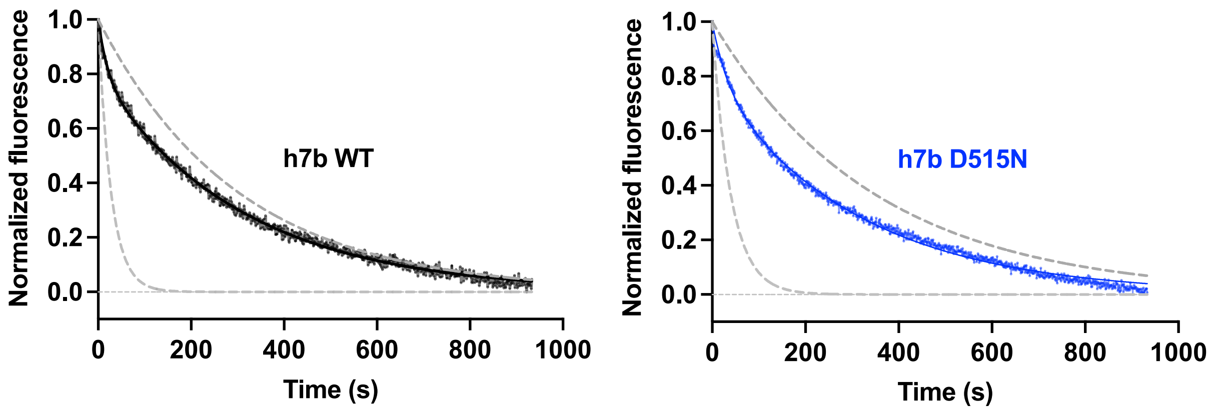

Figure S2. Representative traces for single mant-ATP turnover experiments. Normalized curves for h7b WT S1 (black) and h7b D515N S1 (blue) were fit to a bi-exponential decay with Y_0 = 1 and plateau = 0. The top dark gray dotted line represents data simulated with a single exponential decay with the average slow rate for each construct, and the bottom light gray dotted line represents data simulated with a single exponential decay with the average fast rate for each construct. Technical replicates: h7b WT n = 10, and h7b D515N n = 8. Data are summarized in Table S4.

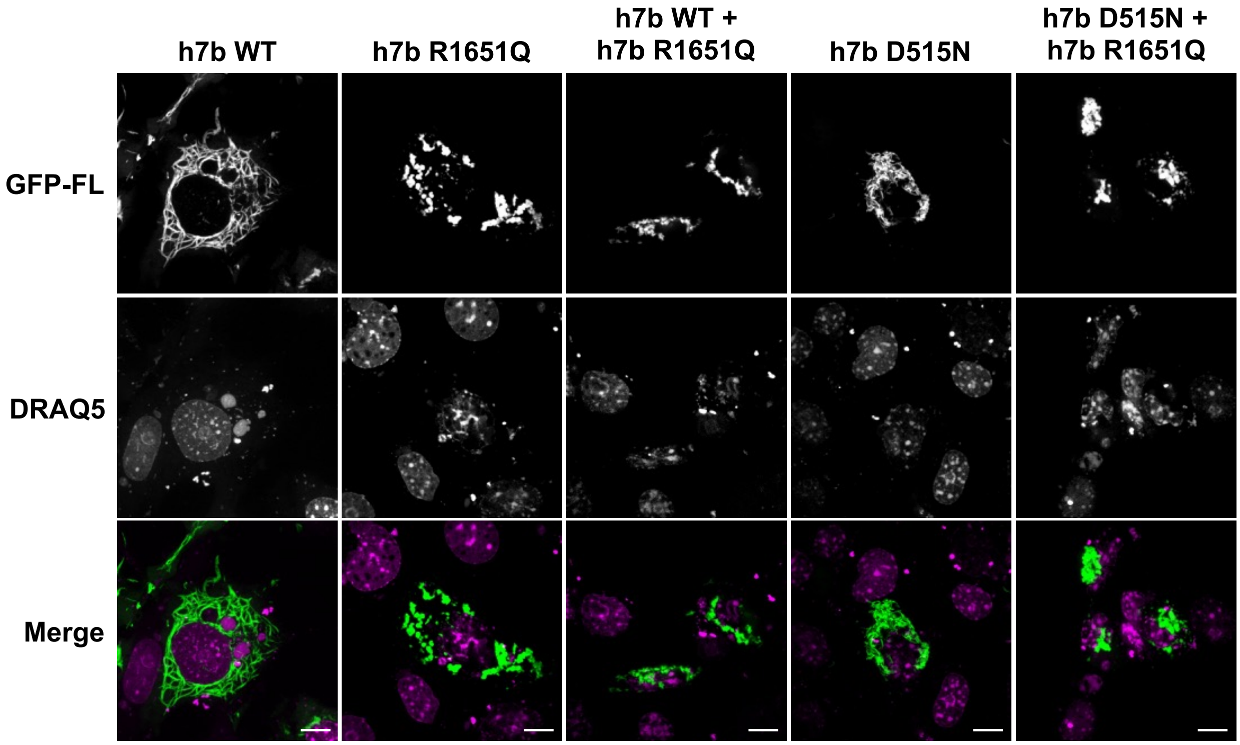

Figure S3. COS-7 cells transfected with GFP-full length (FL) constructs. Representative images for WT alone, R1651Q alone, WT + R1651Q, D515N alone, and the D515N/R1651Q double mutant are shown. All images were taken 18-22 hours after transfection. Cells were stained with DRAQ5 nuclear stain to identify individual cells. Scale bar represents 10 μm.

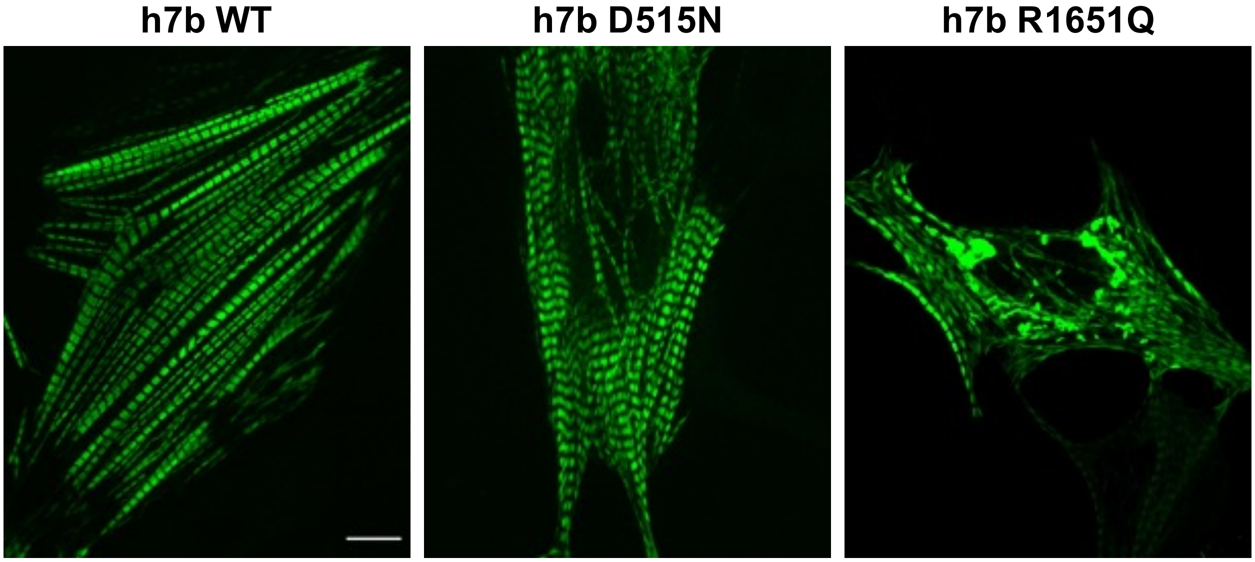

Figure S4. Neonatal Ventricular Rat Myocytes (NRVMs) electroporated with GFP-tagged human MYH7b constructs (MYH7b WT full length, MYH7b D515N full length, and MYH7b R1651Q rod). Representative images for each electroporation are shown. Scale bar represents 10 μm.

Table S1. Actin-activated ATPase results summary

Protein	k_{cat} (s^{-1})	k_{cat} SD (s^{-1})	K_M (μM)	K_M SD (μM)	Biological n (purifications)	Technical n (curves)
h7b WT	0.80	0.08	35.9	9.8	6	9
h7b D515N	0.78	0.15	38.7	27.4	6	9

*Statistical significance ($p < 0.05$ by two-way t-test)

Table S2. Stopped-flow kinetics results summary

Protein	k_{+2} (s^{-1})	k_{+2} SD (s^{-1})	K_1k_{+2} ($\mu M^{-1}s^{-1}$)	K_1k_{+2} SD ($\mu M^{-1}s^{-1}$)	$1/K_1$ (μM)	$1/K_1$ SD (μM)	K_{ADP} (μM)	K_{ADP} SD (μM)	Technical n (curves)
h7b WT	520.7	7.0	6.7	1.4	79.8	17.7	120.7	29.5	2
h7b D515N	590.2	46.6	7.6	0.6	78.5	12.5	81.3	19.5	2

*Statistical significance ($p < 0.05$ by two-way t-test)

Table S3. *in vitro* motility results summary

Protein	Velocity (μm/s)	Velocity SD (μm/s)	Biological n (purifications)	Technical n (movies)	# Filaments
h7b WT	0.597	0.057	3	12	242
h7b D515N	0.716*	0.060	3	12	207

*Statistical significance ($p < 0.05$ by two-way t-test)

Table S4. Single ATP turnover results summary

Protein	% DRX (fast)	% SRX (slow)	% DRX and SRX SD	k_{fast} rate (s^{-1})	k_{fast} rate SD (s^{-1})	k_{slow} rate (s^{-1})	k_{slow} rate SD (s^{-1})	Biological n (purifications)	Technical n (curves)
h7b WT	17.1	82.9	3.7	0.0361	0.0171	0.00334	0.00066	4	10
h7b D515N	27.6*	72.4*	9.3	0.0252	0.0129	0.00287	0.00043	3	8

*Statistical significance ($p < 0.05$ by two-way t-test)

Table S5. COS-7 cell phenotype scoring

Transfection	h7b WT	h7b WT	h7b WT	h7b WT	h7b R1651Q	h7b R1651Q	h7b R1651Q	h7b WT + h7b R1651Q	h7b WT + h7b R1651Q	h7b WT + h7b R1651Q
Needles	32	37	37	23	4	1	2	2	4	25
Aggregates	0	0	0	0	45	25	30	24	31	20
Total Cells	32	37	37	23	49	26	32	26	35	45
